# A 100 mg/kg Dose of Naringenin as an Anti-Obesity Agent for Eight Weeks Exerts No Apparent Hepatotoxic or Nephrotoxic Effects in Wistar Rats

**DOI:** 10.3390/foods14234083

**Published:** 2025-11-28

**Authors:** Gabriela López-Almada, J. Abraham Domínguez-Avila, Gustavo A. González-Aguilar, Rosario Maribel Robles-Sánchez, Norma Julieta Salazar-López

**Affiliations:** 1 Facultad de Medicina y Nutrición de Mexicali, Universidad Autónoma de Baja California, Dr. Humberto Torres Sanginés, Centro Cívico, Mexicali 21000, BCN, Mexico; 2 SECIHTI—Centro de Investigación en Alimentación y Desarrollo A.C., Carretera Gustavo Enrique Astiazarán Rosas No. 46, Col. La Victoria, Hermosillo 83304, SO, Mexico; abrahamdominguez9@gmail.com; 3 Centro de Investigación en Alimentación y Desarrollo A.C., Carretera Gustavo Enrique Astiazarán Rosas No. 46, Col. La Victoria, Hermosillo 83304, SO, Mexico; 4 Departamento de Investigación y Posgrado en Alimentos, Universidad de Sonora, Blvd. Luis Encinas y Rosales Col. Centro. A.P., Hermosillo 83000, SO, Mexico

**Keywords:** Naringenin, metabolism, safety, liver, kidney, electrolytes

## Abstract

Naringenin (NAR) is a naturally occurring flavanone characteristic of citrus fruits and other foods whose anti-obesity effects have been reported. As a dietary xenobiotic, it is metabolized and excreted mainly by the liver and kidneys, respectively. Since an organism does not normally consume pure phenolic compounds, there are concerns about its safety when administered as such. The present work reports an analysis on the safety of consuming NAR as an anti-obesity agent (100 mg/kg body weight) alongside a Western diet (WD) during an eight-week period, according to various serum biochemical markers of liver and kidney function in Wistar rats. Blood samples were analyzed to determine liver function, including enzyme activity (ALT, AST, GGT, and ALP), bilirubin, and albumin. Biochemical markers of kidney function were urea, blood urea nitrogen (BUN), creatinine, uric acid, and electrolytes. Results show that a 100 mg/kg oral dose of NAR for eight weeks exerted no apparent hepato- or nephrotoxicity, suggesting a suitable safety profile at said dose, since all variables analyzed remained within normal reference limits in NAR-treated animals. Urea, BUN, and ALP showed significant differences between the WD and the control group fed a basal diet (BD), although this was independent of NAR (*p* < 0.05, WD and WD + NAR vs. BD and BD + NAR), suggesting that diet played a role. The data support the previously reported hepatoprotective effects of NAR and suggest a favorable safety profile. Altogether, the findings indicate that pure NAR may be safe at the dose employed and during the analyzed time period, which further supports the need for clinical studies to validate its application in human consumers.

## 1. Introduction

Obesity is a chronic disease that affects people of all ages across the world and is known to be a significant public health problem [1]. The excessive accumulation of adipose tissue plays a central role in its detrimental consequences, including the development of associated non-communicable diseases, which contribute to decreasing quality of life and life expectancy [2]. Obesity is multifactorial, but diet is known to have a central role [3]; for example, consuming a Western diet (WD) has been strongly associated with its development and progression [4], particularly due to its macronutrient composition. The WD is specifically characterized by its high content of processed and refined foods, added sugars, and red meats, as well as saturated and *trans* fats. On the other hand, it is also typically low in fiber, fruits, vegetables, whole grains, and, consequently, leads to a low exposure to health-promoting bioactive compounds [5]. These low-quality nutrients contribute to altered processes of chronic inflammation, oxidative stress, and lipotoxicity that can damage cellular components and give rise to the development of chronic non-communicable diseases [6].

In contrast to the WD, a healthy dietary pattern is characterized by being rich in foods that contain bioactive compounds, such as flavonoids, that have been shown to exert an anti-obesity effect. The mechanisms of action of these bioactive compounds include preventing and/or countering some of the underlying dysregulated molecular processes characteristic of obesity, such as adipogenesis, inflammation, and oxidative stress [7].

Naringenin (NAR, 5,7,4′-trihydroxyflavanone) is the aglycone form of naringin (5,7,4′-trihydroxyflavanone-7-rhamnoglucoside). It is a naturally occurring flavanone characteristic of citrus fruits like grapefruit, orange, and lemon, as well as dates, tomatoes, and others, whose health-promoting effects have been recently studied [8,9,10]. The literature contains significant evidence for a range of biological activities, including anti-obesity effects documented in both human and rodent models when ingested from natural and synthetic sources [11,12,13]. Some of these activities include anti-dyslipidemic [14], antioxidant, anti-inflammatory [15], and hepatoprotective effects [16,17]. Some novel mechanisms have also been recently reported, including a potential satiety-promoting effect, according to changes exerted on satiety-related hormones when NAR is orally administered in a murine model [9]. Some of the beneficial effects of NAR may be the result of its ability to modulate hormonal responses, countering lipogenic and inflammatory signaling pathways, and its antioxidant capacity that acts against systemic oxidative stress [17,18,19]. Such important information is promising but suggests the need for further research to take into consideration the safety of consuming NAR as an anti-obesity agent in its pure form.

Analyzing the potential hepatotoxicity and nephrotoxicity of NAR is an essential step in validating it as a safe and effective anti-obesity agent, which may then support its subsequent application in human and clinical studies. NAR, as many other dietary compounds, is classified as a dietary xenobiotic; orally consumed xenobiotics reach the small intestine where they are absorbed into circulation and transported to different organs, including the liver. Both the small intestine and liver express various phase I and II enzymes that metabolize—or biotransform—most dietary xenobiotics in order to neutralize any potential toxicity and increase their solubility to facilitate renal excretion [20]. The liver is the primary organ for all xenobiotic metabolism and is therefore exposed to the dietary xenobiotics that an organism consumes orally, which makes it highly susceptible to drug-induced liver injury. Moreover, its role in glucose and lipid metabolism also makes it a prime target of the effects of the organism’s diet, which predisposes it to diseases like metabolic dysfunction-associated fatty liver disease (MAFLD) [21]. The prominent physiological role of the liver allows for clear analyses to evaluate potential injury or toxicity; traditional serum biomarkers like alanine aminotransferase (ALT), aspartate aminotransferase (AST), gamma-glutamyl transferase (GGT), and bilirubin are often measured [22] in order to determine the effects of a potentially hepatotoxic compound. A recent meta-analysis reports various metabolic improvements when eight phenolic compounds were administered (including NAR) in clinical trials of nonalcoholic fatty liver disease, in addition to minimal signs of toxicity according to liver enzymes and other biomarkers, although the authors point out that the safety of NAR and other compounds still requires further validation [23].

Serum albumin is a widely used biochemical parameter that simultaneously provides insight into liver and kidney function. Albumin is synthesized by the liver and is commonly used in the assessment of chronic liver disease. As the primary protein in blood, a decrease in albumin via urinary excretion could indicate kidney damage, typically in the context of kidney injury or disease. Low levels of albumin are often associated with chronic malnutrition [24], systemic inflammation, cirrhosis, and other pathologies involving fluid retention like ascites [25], although it has also been used as a marker and predictor of acute kidney injury [26].

In addition to their basal metabolic functions, the kidneys play a crucial role in the clearance of dietary xenobiotics and their metabolites, mediating their elimination from blood into urine, while also contributing to electrolyte regulation. Thus, dietary xenobiotics like NAR and its metabolites may directly induce cellular damage within this organ [27], which merits attention. Nephrotoxicity can be partially evaluated through classical biomarkers of kidney function, such as urea, blood urea nitrogen (BUN), creatinine, and electrolytes. Changes in serum electrolyte concentration have also been associated with dietary factors, such as a high-fat diet, which can cause renal injury due to increased oxidative stress and inflammation [28,29]. The effects of consuming phenolic compounds from different sources are generally regarded as safe for kidney health; in fact, they may even be nephroprotective [30], although NAR still requires particular attention to validate its safety profile.

The aim of the present work was to analyze the potential hepatotoxic and nephrotoxic effects of administering a 100 mg/kg oral dose of NAR over eight weeks as an anti-obesity agent in rats. Due to the previously reported success of NAR in modulating obesity-related parameters, important questions arise about its safety, since any adverse effects may limit its role in subsequent studies, particularly those involving human participants. The data aims to address concerns about the systemic effects of NAR which may compromise liver or kidney function. This contributes to an integral approach for a better understanding of its safety profile and supports its continued study as a potential anti-obesity agent.

## 2. Materials and Methods

### 2.1. Ethical Statement

The experimental protocol was reviewed and approved by the University of Sonora Research Ethics Committee (CEI-UNISON No. 20/2023, approved on the 23 August 2023). All research was conducted in accordance with the 3Rs and with Mexican regulation NOM-026-ZOO-1999 regarding the care and use of laboratory animals, as well as all other applicable institutional and national regulation.

### 2.2. Study Design

Male Wistar rats (8 weeks of age, 278 ± 4 g) were obtained from the Department of Food Research and Graduate Studies of the University of Sonora (Hermosillo, Sonora, Mexico). Rats were randomly divided into four groups (*n* = 6/group), housed in individual metabolic cages under controlled conditions (25 °C; 40–70% humidity; 12 h light/dark cycles), and had free access to basal rodent diet and water. After one week of acclimatization under said conditions, each group was fed a different experimental diet [9]: Group 1 (BD) was fed a basal diet with 15% energy from lipids, 64% from carbohydrates, and 20% from protein; Group 2 (BD + NAR) was fed the same basal diet in addition to NAR; Group 3 was fed a Western diet (WD) with 36% energy from lipids, 47% from carbohydrates, and 16% from proteins [31,32]; in addition, 20% fructose was added to the animals’ drinking water, with the aim of imitating the high consumption of sugary drinks (sodas, juices, etc.) that is characteristic of the Western diet. Group 4 (WD + NAR) was fed the WD in addition to NAR. Vitamins and minerals were also added to all diets in order to maintain an appropriate micronutrient concentration. Supplementary Table S1 describes the detailed composition of the experimental diets. Diets were not isocaloric, and their energy density (kcal/g) does not include the fructose in the drinking water; BD and BD + NAR had 4.06 kcal/g, while WD and WD + NAR had 4.70 kcal/g. Energy was determined according to the following equivalences: carbohydrates: 4 kcal/g; lipids: 9 kcal/g; protein: 4 kcal/g; and fiber (cellulose): 2 kcal/g, as specified by the FDA’s nutrition labeling regulations [33]. Natural-grade NAR of 98% purity was used (W530098, Sigma-Aldrich, St. Louis, MO, USA); the dose administered (100 mg/kg body weight) was determined based on previous reports [12]. NAR was first dissolved in 0.5% carboxymethylcellulose (CMC) as vehicle and administered by oral gavage once daily at the same time for eight weeks. Groups without NAR were administered an equivalent volume of 0.5% CMC by oral gavage.

After eight weeks of consuming said experimental diets, the animals were fasted and anesthetized (intraperitoneal pentobarbital dose of 60 mg/kg, Pisa, Mexico). Once a complete absence of reflexes was confirmed, a cardiac puncture was performed, from which blood was collected into serum-separating tubes and centrifuged (1500× *g*, 10 min). Serum was obtained and the biochemical analyses described in the following section were performed on it.

### 2.3. Biochemical Analyses

Hepatic and renal function analyses were determined in serum samples by commercial kits (Randox, Crumlin, Antrim, UK), following the supplier’s instructions. Hepatic function tests included enzyme activity of aspartate aminotransferase (AST) (Cat. No. AS8306), alanine aminotransferase (ALT) (Cat. No. AL8301), gamma-glutamyl transferase (GGT) (Cat. No. GT8320), and alkaline phosphatase (ALP) (Cat. No. AP8302). The quantification of AST is based on the reaction between α-oxoglutarate and l-aspartate to form l-glutamate and oxaloacetate. For ALT, α-oxoglutarate reacts with l-alanine to form l-glutamate and pyruvate. NADH is consumed in both reactions, and its decrease was monitored at 340 nm using a FLUOstar^®^ Omega 5.10 (Ortenberg, Germany) microplate reader in order to quantify enzyme activity. The quantification of GGT is based on the reaction between l-γ-glutamyl-3-carboxy-4-nitroanilide and glycylglycine, which yields l-γ-glutamylglycylglycine and 5-amino-2-nitrobenzoate; the increase in absorbance of 5-amino-2-nitrobenzoate was read at 405 nm. For ALP, p-nitrophenylphosphate reacts with H_2_O to yield phosphate and p-nitrophenol; the absorbance of the latter is read at 405 nm. Results of enzyme activity were expressed in U/L, where one unit is defined as the amount of enzyme that catalyzes the conversion of 1 µmol of substrate/min, according to the aforementioned reactions.

Serum albumin (Cat. No. AB3800) was also analyzed and expressed in g/dL, while total bilirubin (Cat. No. BR4061) was expressed in mg/dL.

Renal function tests included urea (Cat. No. UR8334), blood urea nitrogen (BUN), creatinine (Cat. No. CR4037), and uric acid (Cat. No. UA8333); results were expressed in mg/dL. BUN was calculated according to Equation (1):

(1) $$BUN\left(\frac{mg}{dL}\right)=\frac{Urea(\frac{mg}{dL})}{2.14}$$

Serum electrolytes were also quantified, specifically, Na^+^ (Cat. No. NA3851), K^+^ (Cat. No. PT8329), Cl^−^ (Cat. No. CL1646), Ca^2+^ (Cat. No. CA 8309), and P (Cat. No. PH8328); results were expressed in mEq/L for Na^+^, K^+^, and Cl^−^ and in mg/dL for Ca^+2^ and P.

### 2.4. Statistical Analysis

Samples were analyzed in duplicate. Statistical analysis was executed using JMP 8.0 software (Cary, NC, USA), through an analysis of variance (ANOVA) and Tukey–Kramer’s test. Results are expressed as mean ± standard error of the mean (SEM). Differences were considered significant when *p* < 0.05.

## 3. Results

The anti-obesogenic effects of NAR in this model have been previously described, where it was shown that it acted on adipose tissue, mainly retroperitoneal, among other relevant actions [9]. Thus, the present work focuses on evaluating the safety of the treatment, as determined by changes in liver and kidney function parameters.

### 3.1. Biomarkers of Liver Function

**Figure 1:**
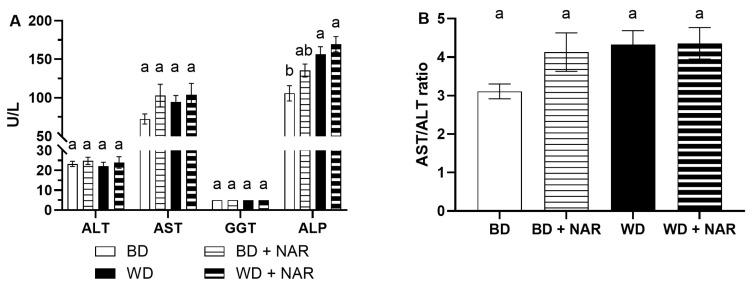
(**A**) Hepatic enzyme activity of alanine amino transferase (ALT), aspartate aminotransferase (AST), gamma-glutamyl transferase (GGT), and alkaline phosphatase (ALP). (**B**) AST/ALT ratio. BD: basal diet; BD + NAR: basal diet and 100 mg/kg of naringenin; WD: Western diet; WD + NAR: Western diet and 100 mg/kg of naringenin. Results are expressed as mean ± SEM of two independent replicates. Different lowercase letters indicate significant differences for each variable, according to an ANOVA and Tukey–Kramer’s test (*p* < 0.05).

Various enzymatic and non-enzymatic biomarkers of liver function were analyzed at the end of the eight-week experimental period. The activities of four liver enzymes (ALT, AST, GGT, and ALP) are shown in Figure 1.

Serum ALT showed no variation between groups, with all of them having values between approximately 22 and 25 U/L (*p* > 0.05). Normal values have been reported between 19.78 and 50.55 U/L [34], 24 and 49 U/L [35], and 20 and 61 U/L [36].

The activities of AST showed minimal variation between groups, although they were not significant (*p* > 0.05). They were in the range of approximately 72–104 U/L. Others have reported normal values of 39.0–111 U/L [36], 94.34–228.28 U/L [34], and 50–96 [35].

Considering the sub-chronic nature of our model, we evaluated the AST/ALT ratio (De Ritis ratio) which indicates the severity of hepatic disease and could assist in establishing acute or chronic liver injury [37]. The results show no significant differences between groups, with values ranging from 3.1 to 4.3, which is consistent with previous findings on enzyme activity.

Activity of GGT remained unchanged between groups, with all of them having similar values of 5.0 U/L and no significant differences between them (*p* > 0.05). Other authors report normal values in the range of 0–6 U/L [36].

The activity of ALP did show significant differences (*p* < 0.05), mainly between the BD and the WD and the WD + NAR groups, while the BD + NAR group had intermediate values that were statistically similar to the other groups’. Values ranged from 105 to 169 U/L, with others reporting normal values in the range of 16–302 U/L [36], 137.35–437.41 [34], and 65–193 U/L [35].

**Figure 2:**
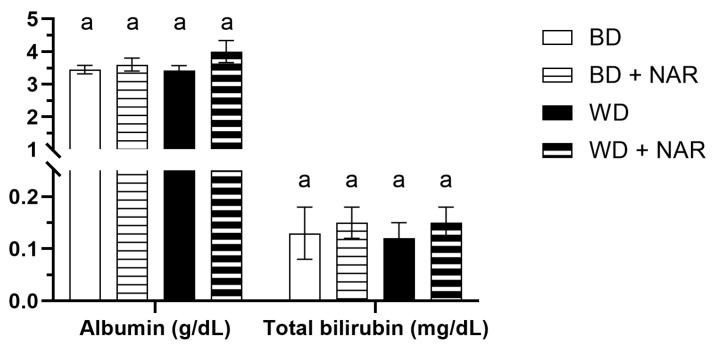
Albumin and total bilirubin concentration. BD: basal diet; BD + NAR: basal diet and 100 mg/kg of naringenin; WD: Western diet; WD + NAR: Western diet and 100 mg/kg of naringenin. Results are expressed as mean ± SEM of two independent replicates. Different lowercase letters indicate significant differences for each variable, according to an ANOVA and Tukey–Kramer’s test (*p* < 0.05).

The concentrations of albumin and bilirubin are shown in Figure 2.

Similar values of albumin were apparent, which ranged from 3.4 to 4.0 g/dL and showed no significant difference between groups (*p* > 0.05). Other authors report normal values in the range of 3.2–4.62 g/dL [34] and 28–53 g/L (equivalent to 2.8–5.3 g/dL) [38].

Total bilirubin also remained stable, with values in the range of 0.12–0.15 mg/dL and no significant differences between groups (*p* > 0.05). Others report normal values in the range of 0.02–0.31 mg/dL [34] and 0.1–0.7 mg/dL [36]. Thus, serum albumin and bilirubin levels remained unaffected by either NAR administration or dietary intervention.

### 3.2. Biomarkers of Kidney Function

**Figure 3:**
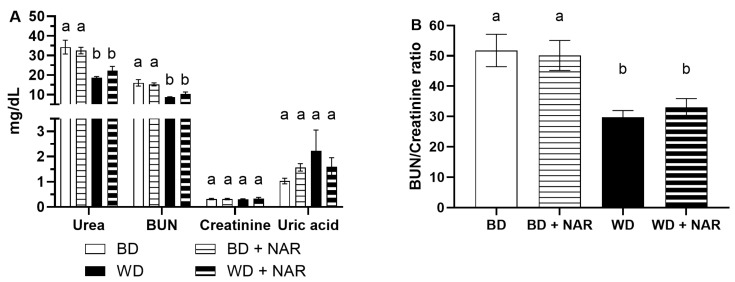
(**A**) Biomarkers of renal function. (**B**) BUN/Creatinine ratio. BUN: blood urea nitrogen (BUN). BD: basal diet; BD + NAR: basal diet and 100 mg/kg of naringenin; WD: Western diet; WD + NAR: Western diet and 100 mg/kg of naringenin. Results are expressed as mean ± SEM of two independent replicates. Different lowercase letters indicate significant differences for each variable, according to an ANOVA and Tukey–Kramer test (*p* < 0.05).

Kidney function was also analyzed at the end of the eight-week experimental period, according to different serum biomarkers. The concentrations of urea, BUN, creatinine, and uric acid are shown in Figure 3.

Significant differences between groups were found for urea (*p* < 0.05), where WD and WD + NAR groups showed significantly lower values, as compared to the BD and BD + NAR groups. Thus, these changes were apparently caused by the administration of the WD, and not by NAR. Regardless of said differences, all values ranged from 18 to 34 mg/dL, which are within normal values reported in the range of 13.57–42.56 mg/dL [34], 18–45 mg/dL [36], and 4.0–9.3 mmol/L (equivalent to 24.0–55.8 mg/dL) [35]. A similar behavior was observed in BUN levels, with the WD and WD + NAR groups showing significantly lower values (*p* < 0.05 vs. BD and BD + NAR). BUN values in all groups ranged from approximately 8 to 16 mg/dL.

Creatinine levels were similar across all experimental groups (*p* > 0.05), with values ranging from approximately 0.30 to 0.33 mg/dL. Others report normal values in the range of 0.21–0.68 mg/dL [34], 0.05–0.65 mg/dL [36], and 31–48 μmol/L (equivalent to 0.35–0.54 mg/dL) [35].

The BUN/creatinine ratio ranged from 30 to 52, showing significant differences between dietary groups. This pattern mirrored that of BUN and urea, with the BD and BD + NAR exhibiting higher ratios and the WD and WD + NAR having lower ones. The BUN/creatinine ratio is commonly used in clinical settings to help determine underlying causes of acute kidney injury and helps in distinguishing between pre-renal and intrinsic etiologies. The main intrinsic cause is acute tubular necrosis and, in this context, drug-induced nephrotoxicity may contribute to renal impairment [39].

Regarding uric acid, there were no significant differences between groups (*p* > 0.05), although the WD group showed a slight but non-significant increase, with observed values from approximately 1.0 to 2.2 mg/dL. Others report normal values in the range of <6.8 mg/dL [40], 0.5–1.5 mg/dL [41], and 12–54 μmol/L (equivalent to 0.20–0.90 mg/dL) [35].

**Figure 4:**
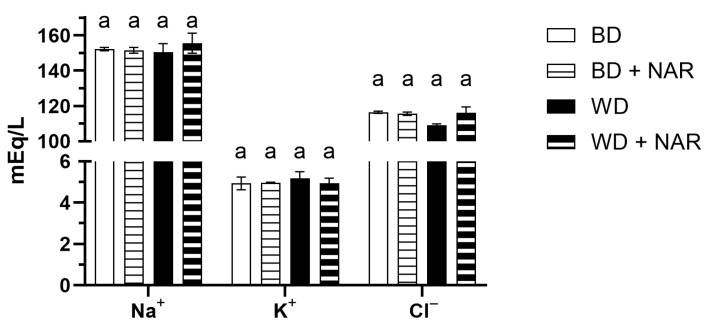
Na^+^, K^+^, and Cl^−^ concentration as biomarkers of renal function. BD: basal diet; BD + NAR: basal diet and 100 mg/kg of naringenin; WD: Western diet; WD + NAR: Western diet and 100 mg/kg of naringenin. Results are expressed as mean ± SEM of two independent replicates. Different lowercase letters indicate significant differences for each variable, according to an ANOVA and Tukey–Kramer’s test (*p* < 0.05).

**Figure 5:**
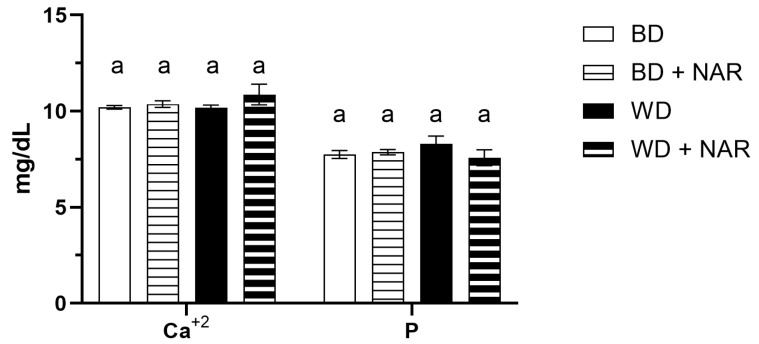
Ca^+2^ and P concentration as biomarkers of renal function. BD: basal diet; BD + NAR: basal diet and 100 mg/kg of naringenin; WD: Western diet; WD + NAR: Western diet and 100 mg/kg of naringenin. Results are expressed as mean ± SEM of two independent replicates. Different lowercase letters indicate significant differences for each variable, according to an ANOVA and Tukey–Kramer’s test (*p* < 0.05).

Electrolyte concentration is shown in Figure 4 (Na^+^, K^+^, and Cl^−^) and in Figure 5 (Ca^+2^ and P).

Na^+^ concentration remained stable across all groups, showing no significant differences (*p* > 0.05). Values ranged from approximately 150 to 155 mEq/L, with other authors reporting normal values between 129 and 156 mEq/L [38], 131 and 142 mEq/L [35], and 135 and 146 mEq/L [36].

K^+^ concentration did not vary between groups (*p* > 0.05) and ranged from approximately 4.9 to 5.1 mEq/L. Other authors report normal values of 4.0–8.0 mEq/L [38], 3.9–6.5 mEq/L [35], and 4.0–5.9 [36].

Cl^−^ concentration remained stable across groups, showing no significant differences (*p* > 0.05). Values ranged from approximately 109 to 116 mEq/L. Others report normal values in the range of 96–107 mEq/L [36] and of 118.67 mEq/L [42].

Ca^+2^ concentration remained unchanged between groups (*p* > 0.05) and ranged from approximately 10.2 to 10.9 mg/dL. Other authors report normal values in the range of 5.3–11.6 mg/dL [36], 2.02–2.48 mmol/L (equivalent to 8.09–9.93 mg/dL) [35], and 5.0–15.0 mg/dL [38].

P concentration was also similar between groups (*p* > 0.05) and ranged from 7.5 to 8.3 mg/dL. Others report normal values in the range of 3.1–12.2 mg/dL [38].

The results therefore suggest that electrolyte homeostasis was not affected by NAR or dietary intervention.

It should be emphasized that serum biochemistry can show significant variation between laboratories according to the particular method, reagents, and equipment used among other variables and, therefore, reference ranges commonly differ between laboratories. This makes it important to interpret data in the context of normal or control groups for each experiment, even more so in animal experimentation, where reference values are not as standardized as those for humans. For example, Houtmeyers et al. [38] compiled data for different variables from multiple sources, which showed considerable differences between them.

## 4. Discussion

The present study focused on the safety profile of pure orally administered NAR in rats at a dose of 100 mg/kg body weight for eight weeks as an anti-obesity agent when given alongside a basal or Western diet. Previous evidence demonstrated a favorable outcome of this treatment [9], potentially due to NAR regulating dyslipidemia and adiposity. Such findings highlight the importance of scrutinizing potential negative effects, including those on the liver and kidneys.

Altered aminotransferase levels reflect hepatocellular injury and necrosis, while the AST/ALT ratio is used as a predictive tool and biomarker of potential drug-induced liver injury (DILI), as well as to diagnose cirrhosis and acute alcoholic hepatitis [37]. Changes in ALP activity suggest injury to the biliary epithelial cells or canalicular membrane. GGT, although present in other tissues, can be used to detect hepatobiliary disease, in particular cholestasis and biliary effects. On the other hand, bilirubin reflects overall liver function, as well as the liver’s excretory capacity and hepatobiliary health [43].

The assessment of serum liver function enzymes AST, ALT, and GGT showed no significant changes, suggesting that the NAR treatment did not exert a negative impact on the liver, which could have resulted in increased enzyme activity. NAR has shown hepatoprotective effects in other experimental models, like in a pyrazinamide (PZA)-induced hepatotoxicity model in rats, in which a 50 mg/kg dose (half of the dose reported in the present study) given for 28 days effectively prevented elevation of serum AST and ALT activities (*p* < 0.05, PZA + NAR vs. PZA) [22]. Similarly, 100 mg/kg of NAR for seven days significantly prevented the increase in hepatic enzymes ALT and AST in a dasatinib-induced liver injury model (*p* < 0.05 vs. damage group) [44].

Regarding ALP, its activity remained within normal values, although an increase was found. This change appears to be due to the WD and not from NAR itself, which may be attributable to hepatic metabolic adaptations associated with diet-induced obesity caused by the WD, which induces steatosis and low-grade inflammation. Serum ALP levels have been associated with fructose intake in rats [45] and metabolic syndrome components in humans [46]; it has even been proposed as a biomarker and predictor of fibrosis in obese patients with nonalcoholic fatty liver disease (NAFLD) [47], showing significant correlation with MAFLD (*p* < 0.001) [48]. The non-significant tendency of higher ALP values observed in NAR-treated groups (BD + NAR and WD + NAR) may be attributable to various factors; for example, since it is not expressed exclusively in the liver, it is possible that NAR may be modulating it in other tissues. In support of this hypothesis, Foster et al. [49] report a hepatoprotective effect and an increase in intestinal ALP exerted by a phenolic-rich beverage (Fuzhuan tea) in rats fed a high-saturated-fat diet and exposed to bacterial endotoxin. Since one activity of ALP is to inactivate bacterial endotoxin by dephosphorylation, among other protective effects on the intestinal barrier, the authors propose that the increase in ALP may be protective. Chen et al. [50] also report a similar ALP increase in the jejunum of pigs when fed a chlorogenic acid-supplemented diet (1000 mg/kg of diet). Due to the observed improvements in growth and performance, the authors propose that increased jejunal ALP (alongside other biomarkers) could have contributed to the digestive and absorptive process of the animals. Given the results reported in the present study and those of other authors, it is possible that a protective effect of NAR was exerted on the liver and small intestine, which was reflected on a slight tendency to increase serum ALP activity within normal values. Since the exact origin of the enzyme was not determined, this remains an open question to verify in subsequent studies.

The liver is the main site of albumin synthesis; changes in its serum concentration may indicate liver dysfunction, typically indicating chronic damage, while losses are also possible due to renal loss in the context of acute or chronic kidney disease. Bilirubin is a waste product derived from heme catabolism; since it is primarily excreted via the bile, higher concentrations may indicate increased heme catabolism and/or biliary obstruction. Both serum albumin and bilirubin levels remained stable, showing no significant changes between groups, regardless of dietary intervention or NAR administration. These findings suggest no apparent signs of dysfunction in the context of hepatic protein synthesis or renal loss. Other authors propose that phenolic compounds may even counter albumin loss; for example, Foroutanfar et al. [51] report a protective effect of punicalagin, a pomegranate-derived phenolic, in rats treated with acrylamide, a potent neurotoxic and hepatotoxic agent. The authors showed that acrylamide induced a significant decrease in serum albumin, which the punicalagin treatment dose-dependently countered. A nephroprotective effect was reported by Almundarij et al. [52] in rats treated with carbon tetrachloride, a potent nephrotoxic, in response to administering phenolic-rich extracts of *Anastatica hierochuntica* L., a medicinal herb. The authors showed that the extracts normalized serum albumin, which was attributed to the treatments restoring renal function by 78–97%. Thus, phenolic compounds and phenolic-containing sources appear to exert hepatoprotective and nephroprotective effects, which contribute to maintaining or normalizing serum albumin in response to different toxic agents. The results from the present study and those in the literature support the biochemical safety of NAR at the administered dose according to serum albumin and bilirubin.

To assess potential toxic effects of NAR on the kidneys, specific biomarkers were analyzed. Urea is produced in the liver as a result of protein metabolism, followed by renal excretion. Creatinine is also a waste product derived from the non-enzymatic breakdown of creatine phosphate in skeletal muscle which is also excreted by the kidneys. Both compounds are therefore commonly used as biomarkers of kidney function and excretory capacity. In this study, urea and BUN showed significant changes but remained within the normal range; the observed differences were in response to the WD and not of NAR. This suggests that these differences are most likely attributable to dietary composition, specifically, the lower protein intake of the WD (16% energy from protein), as compared to the BD (20% energy from protein). Additionally, creatinine concentrations were found to be within normal limits, which indicates that renal excretory function and metabolic homeostasis were not compromised. Our results coincide with those of Kahramanoğullari et al. [53], in which administering 100 mg/kg of NAR for 20 days did not change plasma urea or creatinine levels; in fact, it prevented their increase in a model of mercury chloride-induced injury (*p* < 0.001 vs. damage group). It also exerted antioxidant effects that protected the kidneys, according to biomarkers like malondialdehyde, glutathione, glutathione-S-transferase, and superoxide dismutase (*p* < 0.001 vs. damage group) [53]. Creatinine also remained stable in response to the diets; interestingly, other authors report that some diets, particularly those with high salt and fat content, have led to changes in serum creatinine concentration [28].

Uric acid tends to increase in response to WD and similar high-fat diets. Hyperuricemia is therefore commonly associated with dyslipidemia, metabolic syndrome, obesity, and obesity-related complications. Obesity is a known risk factor for hyperuricemia, since uric acid synthesis may increase in adipose tissue, and has also been partially associated with obesity-related changes in gut microbiota [54]. Other authors report that serum uric acid increases in response to a high-fat, high-sucrose diet; for example, Yustisia et al. [55] fed a high-fat high-fructose diet (HFHFD) to Wistar rats for eight weeks, a similar timeline to the one in the present study. The authors showed that the HFHFD groups showed a 2.87-fold increase in serum uric acid, as compared to the standard chow group (*p* < 0.05). This effect is primarily due to hepatic fructose metabolism which, when consumed in large amounts, is known to strongly induce de novo uric acid synthesis [56]. Hyperuricemia can also result from altered kidney function and reduced uric acid clearance. Although hyperuricemia was not found in the present study, the WD group showed a non-significant increasing tendency (*p* > 0.05); this is compatible with the expected effects of diet, which may reveal significant changes after longer experimental periods; however, they were not apparent during the timeframe analyzed. Others report that NAR may even reduce uric acid concentrations via urine excretion [19].

The assessment of serum electrolyte concentrations is fundamental to determining renal function and evaluating the safety profile of dietary xenobiotics. Our findings indicate no apparent changes in Na^+^, K^+^, Cl^−^, Ca^+2^, or P. Reports of the effect of NAR on serum electrolytes are very limited; one study evaluated serum urea, creatinine, Na^+^, and K^+^ levels following oral administration of NAR at 20 mg/kg/day for 10 days in an acute cisplatin nephrotoxicity model and found normal kidney function in response to NAR [57]. Although our findings and those of Badary et al. [57] suggest kidney safety according to electrolyte homeostasis, additional evidence is needed.

**Figure 6:**
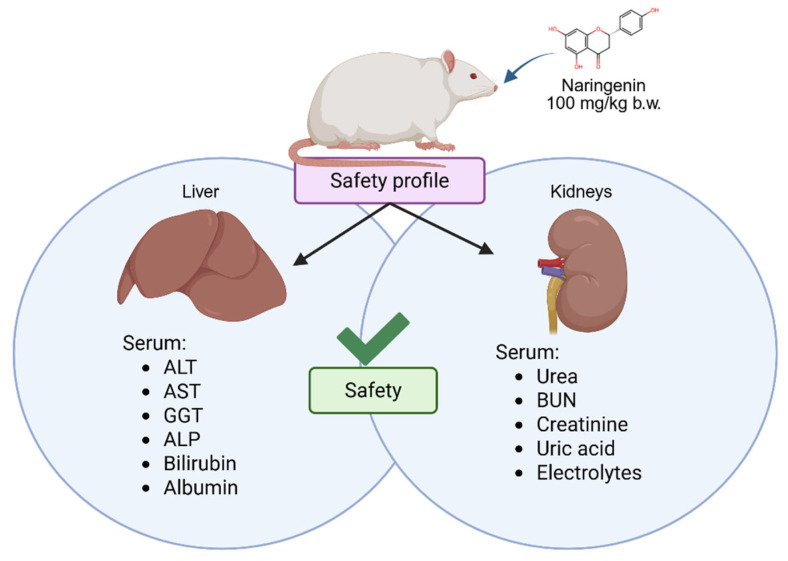
An oral naringenin (NAR) dose of 100 mg/kg for eight weeks was administered to rats alongside a basal diet and a Western diet. No apparent toxicity was found, according to alanine aminotransferase (ALT), aspartate aminotransferase (AST), gamma-glutamyl transferase (GGT), alkaline phosphatase (ALP), bilirubin, albumin, urea, blood urea nitrogen (BUN), creatinine, uric acid, and electrolytes.

Altogether, the data reported here suggests that NAR, at the administered dose and timeframe, exerts no apparent signs of liver or kidney toxicity and may even be hepatoprotective and nephroprotective, as summarized in Figure 6. These findings are promising and support its continued study to improve health in different models. The safety of NAR at this dose aligns with what is often seen with other flavanones, such as hesperidin, which typically require extremely high doses in the order of various g/kg of body weight to reach toxic or lethal levels [58], which further supports their favorable safety profiles.

The present research focused on evaluating a 100 mg/kg of body weight NAR dose as an anti-obesity agent. This particular dose was chosen due to two main factors; first, this dose (as well as higher ones) has shown improvements in obesity biomarkers in previous investigations [12]. Second, it can be potentially extrapolated into a reasonable amount for human beings; some studies tend to focus on very high doses that, although potentially bioactive, would require humans to consume excessively high doses per day. Although an anti-obesity effect could be found with these high doses, the benefits of consuming such a treatment would be negated by potential organ toxicity.

It should nevertheless be emphasized that these safety findings were obtained from a rodent model. Animal and human physiology has some inherent differences due to weight, body surface area, metabolic activity, etc., and if this apparently safe dose were to be extrapolated to a human, there are various approaches for achieving this. The Food and Drug Administration (FDA) suggests a conversion factor of 6.2 to calculate a human equivalent dose (HED) from a rat [59]; thus, 100 mg/kg/d ÷ 6.2 = 16.129 mg/kg/d HED, which is approximately 967.74 mg/d for a 60 kg adult. Moreover, a safety factor of 10 is usually applied to this value, which results in a final HED of 1.613 mg/kg/d HED or approximately 96.78 mg/d for a 60 kg adult.

As previously mentioned, there are few studies focusing on the safety and/or effects of NAR. Rebello et al. [60] report a study on the safety and pharmacokinetic profile of NAR in adults who consumed a single dose of 150, 300, 600, or 900 mg of NAR or placebo and report no relevant adverse effects. In fact, they argue in favor of the tolerability of said doses, with a proposed regimen of 300 mg twice daily (600 mg/d total) since the metabolism of NAR is rapid and its metabolites are cleared in approximately 24 h. Although the study of Rebello et al. [60] only evaluated a one-time dose, our findings are in line with theirs, where a similar dose was used in rats (adjusted to the animals’ body weight) for a significantly longer period showed no apparent liver or kidney toxicity. Future experiments could therefore consider NAR doses of 100–1000 mg/d for adult consumers.

### Limitations

While the findings reported in the present work provide valuable insights, there are certain limitations. For example, a more in-depth toxicokinetic study could also consider histopathological evaluation of the kidneys and measurements of urine output. Kidney function was assessed using biomarkers like urea, BUN, and creatinine, which may not fully reflect early and ongoing changes in kidney function during an eight-week experimental period, and it could be too early to detect a potential reduction in glomerular filtration rate. Other more sensitive biomarkers may provide a more rigorous evaluation of renal status [61]. The same applies to the traditional serum biomarkers of liver function, which lack specificity for liver injury, considering that they may increase due to conditions like muscle or cardiac injury or other extrahepatic pathologies; novel biomarkers may therefore strengthen the present evidence [43]. Different doses, timeframes, and analyses on other organs could also provide valuable information regarding the systemic safety of NAR in an animal model in order to support its evaluation in humans. Finally, this study included only male rats in order to minimize changes associated with possible hormonal fluctuations in females. Previous research has identified that female animals exhibit greater resistance to obesity-induced renal and hepatic dysfunction compared to males; thus, future studies could include evaluations in male and female rats, which could show sex-specific effects that have been previously reported but were not considered in the present work [62,63,64].

## 5. Conclusions

The present work reports that an oral 100 mg/kg body weight dose of naringenin (NAR) administered daily for eight weeks as an anti-obesity agent shows no apparent signs of liver or kidney toxicity, according to classical serum biomarkers of organ function. Hepatic enzyme activity, AST/ALT ratio, albumin, bilirubin, urea, BUN, creatinine, BUN/creatinine ratio, uric acid, and electrolytes (Na^+^, K^+^, Cl^−^, Ca^+2^, and P) were analyzed and shown to be within expected values; no notable differences were found that could indicate organ toxicity due to consuming NAR. Such findings contribute significant evidence regarding the safety profile of NAR as an anti-obesity agent, which might support its future application in the clinic. As a significant public health problem, using natural compounds to treat and/or prevent obesity merits further consideration; moreover, focusing on ones that are already safely consumed in citrus fruits is particularly promising. The administered dose could be theoretically extrapolated in order to validate the findings reported in the present document in human participants, with the goal of promoting the use of natural, effective, and safe anti-obesogenic compounds. Finally, the authors acknowledge that various improvements could be made to the present work in order to obtain more in-depth data, for example, administering additional doses for longer periods of time, performing histopathological analyses on the liver and kidneys (and potentially other organs), and analyzing additional serum metabolites; such experiments may reveal signs of toxicity that were not detected with the reported data, or potential toxicity on other organs. This could be considered for future works in order to effectively translate basic research in animals into clinical studies that can benefit human health.

## Data Availability

The original contributions presented in this study are included in the article/Supplementary Material. Further inquiries can be directed to the corresponding author.

## Supplementary Materials

The following supporting information can be downloaded at https://www.mdpi.com/article/10.3390/foods14234083/s1: Table S1. Composition of the experimental diets (%).

## Abbreviations

The following abbreviations are used in this manuscript:

WD	Western diet
ALT	Alanine aminotransferase
AST	Aspartate aminotransferase
GGT	Gamma-glutamyl transferase
ALP	Alkaline phosphatase
BUN	Blood urea nitrogen
BD	Basal diet
MAFLD	Metabolic dysfunction-associated fatty liver disease
CMC	Carboxymethylcellulose
DILI	Drug-induced liver injury
NAFLD	Nonalcoholic fatty liver disease
HFHFD	High-fat high-fructose diet
HED	Human equivalent dose
