# Supplementary material for: A 100 mg/kg Dose of Naringenin as an Anti-Obesity Agent for Eight Weeks Exerts No Apparent Hepatotoxic or Nephrotoxic Effects in Wistar Rats
Source: Foods. 2025 Nov 28;14(23):4083. doi: 10.3390/foods14234083 (PMC12692467; doi:10.3390/foods14234083)
Supplement: Supplementary file 1 [file foods-14-04083-s001.zip › foods-3966904-supplementary.pdf]

A 100 mg/kg dose of naringenin as an anti-obesity agent for eight weeks exerts no apparent hepatotoxic or nephrotoxic effects in Wistar rats

Gabriela López-Almada, J. Abraham Domínguez-Avila, Gustavo A. González-Aguilar, Rosario Maribel Robles-Sánchez, Norma Julieta Salazar-López*

Supplementary Materials

Table S1. Composition of the experimental diets (%).

**Component**	**BD**	**WD**
Casein	20	19
Lard	0	16
Soybean oil	7	3
Starch	44.75	23
Maltodextrin	13.2	7
Sucrose	5	23.95
Fiber	5	3
Methionine	0.3	0.3
Choline	0.25	0.25
Vitamins	1	1
Minerals	3.5	3.5
Fructose	-	20% (dissolved in the drinking water)
Energy (Kcal/g)	4.06	4.70 *

BD: basal diet; WD: Western diet. Kcal were determined based on the following equivalences: carbohydrates: 4 kcal per 1 g; lipids: 9 kcal per 1 g; protein: 4 kcal per 1 g; fiber (cellulose): 2 kcal per 1 g. As described by López-Almada et al. [9]. * The calculation of kcal/g does not include the fructose included in the water.
